# The restriction impacts of the Type III restriction-modification system on the transmission dynamics of antimicrobial resistance genes in *Campylobacter jejuni*

**DOI:** 10.3389/fmicb.2025.1496275

**Published:** 2025-07-17

**Authors:** Yu Qiu, Pengbo Guo, Hui Tian, Ye Zhou, Hongling Wen, Hao Liang

**Affiliations:** ^1^Department of Health Inspection and Quarantine, School of Public Health, Shandong University, Jinan, China; ^2^Department of Pathogen Biology, School of Clinical and Basic Medical Sciences, Shandong First Medical University and Shandong Academy of Medical Sciences, Jinan, China; ^3^Department of Radiation Oncology, Qilu Hospital of Shandong University, Cheeloo College of Medicine, Shandong University, Jinan, China

**Keywords:** *Campylobacter jejuni*, restriction-modification system, antibiotic resistance genes, transmission dynamics, drug resistance

## Abstract

**Introduction:**

The spread of antibiotic resistance genes among *Campylobacter jejuni* (*C. jejuni*) is a serious problem, and the effects of the restriction-modification (R-M) system on the transmission dynamics of these genes in *C. jejuni* remain poorly understood.

**Materials and methods:**

Complete genome sequences of *C. jejuni* strains were extracted from the BV-BRC database until March 25, 2024. The phylogenetic and the resistance analysis were used to analyze the distribution of resistance genes in *C. jejuni*. The impacts of the R-M systems on the AMR genes transmission between *C. jejuni* strains and the possible mechanisms were explored through recombination, pangenome and mobile genetic elements analysis.

**Results:**

*C. jejuni* strains carrying the Type III R-M system have a significantly lower number of antimicrobial resistance (AMR) genes compared to strains without this system (*p* < 0.0001), with covariance value being −0.0526. The recombination analysis also shows that the median number of the number of AMR genes in the strains not possessing the Type III R-M system increases by 19.38% compared to strains carrying that system (*p* < 0.0001). We also find that the horizontal gene transfer frequency might have limited relationship with the Type III R-M system in *C. jejuni* through pangenome and mobile genetic elements analysis.

**Conclusion:**

Our research indicates that the Type III R-M system might restrict the transmission of AMR genes potentially by affecting recombination in *C. jejuni*, which provides a theoretical basis for addressing the drug resistance problem.

## 1 Introduction

*Campylobacter jejuni* (*C. jejuni*) is one of the leading bacterial pathogens causing foodborne diarrhea worldwide (Igwaran and Okoh, 2019; European Food Safety Authority, European Centre for Disease Prevention and Control, 2023). This pathogenic bacterium can also trigger extraintestinal infections such as bacteremia (Graham et al., 2024), myocarditis (Jiffry et al., 2023), in more severe cases, irritable bowel syndrome (Takakura et al., 2022) and Guillain-Barré syndrome (Finsterer, 2022). Antibiotics should be considered when *C. jejuni* infection is severe. However, with the misuse of antibiotics in human and veterinary medicine, *C. jejuni* has become increasingly resistant to antimicrobial agents used in animals and clinical settings. Despite several years of active surveillance of *C. jejuni* and reduced antibiotics usage in livestock production around the world, antimicrobial resistance (AMR) in *C. jejuni* remains a great public health challenge globally (Devi et al., 2019; van Vliet et al., 2022; Gao et al., 2023). One report has indicated that AMR markers were detected in 53% of *C. jejuni* isolates in the UK and US from 2001 to 2018 (van Vliet et al., 2022).

Homologous recombination is a genetic process in which DNA molecules exchange genetic material between two similar or identical DNA sequences, playing a significant role in the evolution of antibiotic resistance in bacteria by facilitating the exchange and integration of genetic material that confers resistance traits. And AMR genes mediating AMR carried by mobile genetic elements (MGEs) in *C. jejuni* could be spread within different bacterial species, exacerbating bacterial resistance to antibiotics (Partridge et al., 2018; Shikov et al., 2023). A mobile florfenicol resistance gene in *C. jejuni*, *fexA*, encodes an original florfenicol efflux pump system, conferring a high level of florfenicol resistance. The florfenicol resistance gene is located within a region featuring the *tet (L)*-*fexA*-*catA*-*tet (O)* arrangement, which has been shown to be transferable among *C. jejuni* population, thereby worsening the florfenicol resistance issue (Tang et al., 2020).

In addressing the significant issue of AMR in *C. jejuni*, it is imperative to investigate not only the transmission processes of resistant bacteria but also the dynamics of AMR gene transfer between *C. jejuni* strains. The entry mechanisms for exogenous DNA into a bacterium encompass transformation, conjugation and transduction, with the restriction-modification (R-M) system functioning as a defense against exogenous genetic material invasion (Ershova et al., 2015; Tao et al., 2022). The R-M system is of great significance for regulating the uptake and integration of exogenous DNA in bacteria. It comprises two active enzymes, restriction endonuclease (REase) which cleaves the target DNA sequences (Pingoud et al., 2014), and methyltransferase (MTase) which methylates the specific DNA sequences to protect them from REase hydrolysis (Oliveira and Fang, 2021; Seong et al., 2021), thus forming a natural barrier against foreign genetic materials invasion. Based on the specific DNA locus recognized, molecular structure and cofactor requirements, the R-M system can be roughly classified into four groups: Type I ~ IV R-M system (Isaev et al., 2021). Type I REases are multisubunit enzymes comprising three functionally distinct components: a sequence recognition subunit, a MTase subunit, and a REase subunit (Loenen et al., 2013). The REases characteristically interact with two cognate recognition sites and induce DNA cleavage at a midpoint between the recognition sites. Type II REases exist as homodimeric or tetrameric complexes and mediate sequence-specific DNA cleavage either within or adjacent to their recognition sites (typically 4–8 bp) (Pingoud et al., 2014). Type III REases consist of two subunits: a DNA recognition/modification subunit and a distinct DNA cleavage subunit. The enzyme interacts with two recognition sites and leads to a break at a location relative to one bound recognition sequence (Rao et al., 2013). Type IV Reases specifically cleave modified DNA (Loenen and Raleigh, 2014). AveC4I, a Type I MTase, might increase drug resistance in *Aeromonas veronii* (Ma et al., 2023). One research also proved that Cj1051c, a typeIIputative R-M enzyme, could drastically reduce the conjugation efficiency of the standard *C. jejuni* strain NCTC 11168 (Zeng et al., 2018). Nevertheless, there are few studies about the impact of the R-M system on the distribution of AMR genes in *C. jejuni* and the underlying mechanisms.

This study aimed to investigate how the R-M system influences the transfer of AMR genes between *C. jejuni* strains to better control the spread of resistance genes. The findings could offer a theoretical foundation for managing the transmission of AMR genes, which holds significant implications for public health.

## 2 Materials and methods

### 2.1 *Campylobacter jejuni* genome

One thousand and ninety-two *C. jejuni* strains with high sequencing quality and complete information isolated from Asia, Europe and North America were selected from the BV-BRC database until March 25, 2024 (Olson et al., 2023). Subsequently, their genome files were extracted for our research (Supplementary Table S1).

### 2.2 Phylogenetic tree

*Campylobacter jejuni* genome files extracted from the BV-BRC database were used to construct the core gene phylogenetic tree. Through Snippy, *C. jejuni* genomes were compared with the genome file of *C. jejuni* strain NCTC11168 to generate a core Single Nucleotide Polymorphism (SNP) alignment. Subsequently, Gubbins was used to remove SNP sites in the recombinant regions to obtain a high-quality core SNP alignment (Croucher et al., 2014). Then, RAxML was used to generate the final phylogenomic tree (Stamatakis, 2014), which could be visualized via the interactive website tvBOT (Xie et al., 2023).

### 2.3 Prediction of the R-M system in the genomes

To obtain the impacts of the R-M system on the dynamics of the transfer of AMR genes, the REase and MTase genes related to R-M system were extracted from the REBASE database (Roberts et al., 2023). Using these genes collected from the REBASE database as reference, we compared the *C. jejuni* genome files downloaded with them to obtain the distribution of R-M system. We created a sample pool for local screening of *C. jejuni* strains with the R-M system. BLAST was performed based on the *C. jejuni* genome files downloaded from the BV-BRC database (Supplementary Table S2). The interactive website tvBOT was used to visualize the distribution of the R-M system in *C. jejuni*.

### 2.4 Prediction of AMR genes in the genomes

Choosing the National Center for Biotechnology Information (NCBI) database as the reference database (Feldgarden et al., 2019), acquired AMR genes in *C. jejuni* genome files were predicted using the ABRicate tool (Supplementary Table S3). The distribution of AMR genes in *C. jejuni* was visualized via tvBOT, and to explore the impacts of the R-M system on AMR genes, RStudio packages including ggplot2, RColorBrewer and ggpubr were used to analyze and visualize the differences in the number of AMR genes between *C. jejuni* with or without the R-M system. The significance was analyzed using the Wilcoxon-Mann–Whitney test.

### 2.5 Homologous recombination analysis

The recombination_predictions.gff file and per_branch_statistics.csv file were both generated from the Gubbins process. The former one was used to visualize the homologous recombination predictions in *C. jejuni* via phandango (Hadfield et al., 2017). The latter one contained summary statistics for the number of recombination events reconstructed onto both the internal nodes and the leaves of the phylogenetic tree and along with the.recombination_predictions.embl file which covered detailed information about recombination predictions, we could calculate the total number of recombination events in one *C. jejuni* strain (Supplementary Tables S4, S5). It was used to analyze the differences of the recombination blocks between *C. jejuni* with or without the R-M system via RStudio packages including ggplot2, RColorBrewer and ggpubr. The significance was analyzed using the Wilcoxon-Mann–Whitney test.

### 2.6 Pangenome analysis

*Campylobacter jejuni* genome files downloaded from the BV-BRC database were initially annotated using Prokka (Seemann, 2014). Subsequently, Pangenome analysis was performed using Roary on the generated GFF files (Page et al., 2015). To access the impacts of the R-M system on the distribution of unique and cloud genes in *C. jejuni*, using RStudio packages including ggplot2, RColorBrewer and ggpubr, we analyzed and visualized the differences of the number of these genes between *C. jejuni* with or without R-M system based on the gene_presence_absence.csv file obtained from the Roary process (Supplementary Table S6). The significance was analyzed using the Wilcoxon-Mann–Whitney test.

### 2.7 Prediction of integrating MGEs

MGEs in *C. jejuni* genomes were predicted using MobileElementFinder with default parameters (Johansson et al., 2020) (Supplementary Table S7). Then, RStudio packages including ggplot2, RColorBrewer and ggpubr were employed to analyze and visualize the differences in the number of MGEs between *C. jejuni* with or without the R-M system. The significance was analyzed using the Wilcoxon-Mann–Whitney test.

### 2.8 Covariance calculation

Covariance is calculated using the following formula: Cov (x, y) in Rstudio. The x is the value of AMR genes or MGEs, and for y, it takes the value 1 when the Type III R-M system is present or 0 otherwise. The covariance value represents the changing trend of two-dimensional random variables. If the value is positive, the changing trend is consistent, or, if not, the changing trend is opposite.

## 3 Results

### 3.1 The impacts of the R-M system on the distribution of AMR genes in *Campylobacter jejuni*

Among *C. jejuni* strains selected for our study, according to the geographical distribution, 387 (387/1,092, 35.44%), 356 (356/1,092, 32.6%), and 349 (349/1,092, 31.96%) strains were isolated from Europe, North America and Asia, respectively. According to the host origin distribution, 397 (397/1,092, 36.45%), 340 (340/1,092, 31.14%), 167 (167/1,092, 15.29%), 91 (91/1,092, 8.33%), 54 (54/1,092, 4.95%), 35 (35/1,092, 3.21%) and 8 (8/1,092, 0.73%) strains were isolated from patients, poultry, livestock, birds, monkeys, the environment and black bears, respectively.

First, we analyzed the distribution of the R-M system in *C. jejuni*. Utilizing the REase and MTase genes related to R-M system extracted from the REBASE database, we constructed a sample pool for local screening of *C. jejuni* strains with the R-M system. BLAST was performed based on the *C. jejuni* genome files downloaded from the BV-BRC database. We found that 1,043 (1,043/1,092, 95.51%), 1,092 (1,092/1,092, 100%), 334 (334/1,092, 30.59%) and 1,080 (1,080/1,092, 98.90%) strains carry the Type I, Type II, Type III, and Type IV R-M systems, respectively. The Type I and Type III R-M system were distributed unevenly in *C. jejuni*.

**Figure 1:**
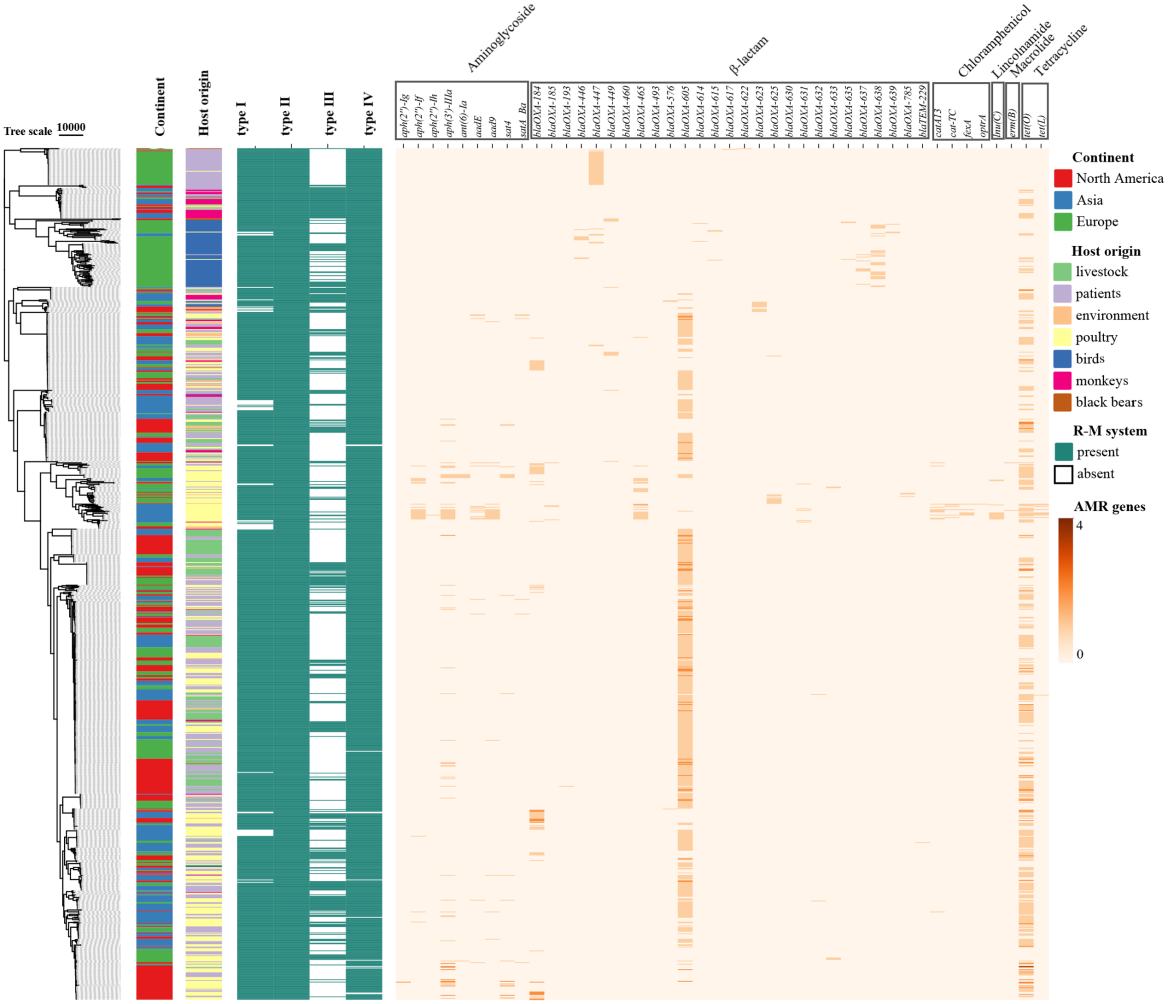
Heatmap of the distribution of the AMR genes in *C. jejuni* based on the phylogenetic tree. The rows in the heatmap represent different *C. jejuni* strains, and the columns represent different AMR genes. The color of the blocks represents the number of the specific AMR gene in one *C. jejuni* strain.

**Figure 2:**
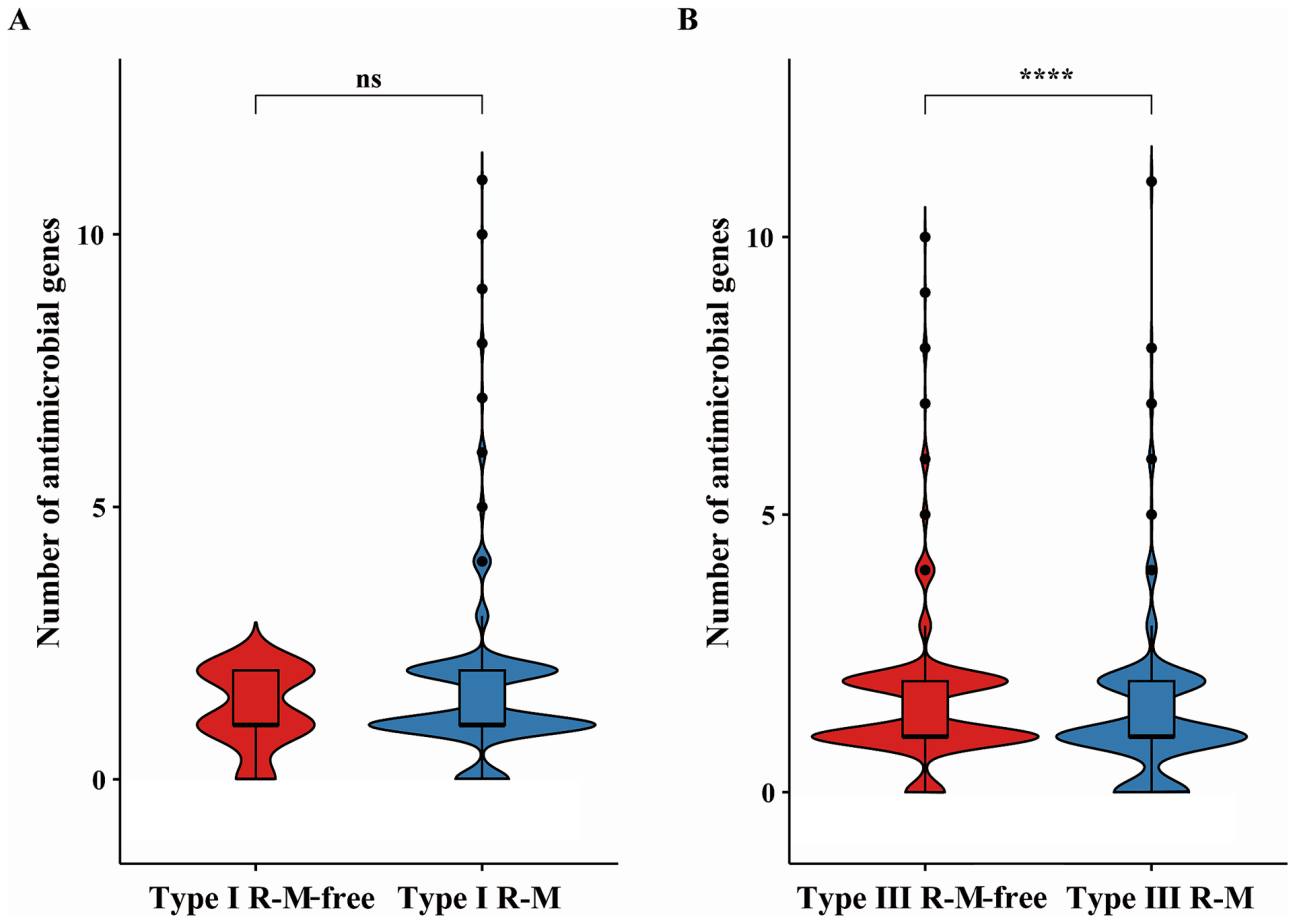
Differences of AMR genes in *C. jejuni* with or without **(A)** the Type I and **(B)** the Type III R-M system. The thicker lines in the box plot represent the median. The box plot’s upper and lower boundaries indicate the 75th (Q3) and 25th (Q1) percentiles, respectively. According to Wilcoxon-Mann–Whitney test, the number of asterisks indicates the significant differences: * indicates *p* ≤ 0.05, ** indicates *p* ≤ 0.01, *** indicates *p* ≤ 0.001, **** indicates *p* ≤ 0.0001, and ns indicates no significant difference.

We explored the distribution of AMR genes and the impacts of the R-M systems on the distribution of AMR genes in *C. jejuni*. Using the ABRicate tool, we identified the acquired AMR genes in *C. jejuni* and quantified them in the genome of the strains (Figure 1). We divided these strains into groups based on the presence of Type I R-M or Type III R-M systems, as well as whether they are R-M-free. We then analyzed the differences in the number distribution of the AMR genes between these groups (Figure 2). The results revealed that *C. jejuni* strains carrying the Type III R-M system had a significantly lower number of AMR genes compared to strains without this system (Wilcoxon-Mann–Whitney test, *p* < 0.0001). To evaluate the directional relationship between the number distribution of AMR genes and the presence of the Type III R-M system, we estimated the covariance value, which shows the relationship between two random variables. The covariance analysis revealed that the covariance value between the Type III R-M group and the Type III R-M-free group was −0.0526, suggesting a negative correlation between the number of AMR genes and the presence of the Type III R-M system. However, there were no significant differences in the distribution of the AMR genes between *C. jejuni* groups with a Type I R-M system or without it (Wilcoxon-Mann–Whitney test, *p* = 0.59).

### 3.2 The existence of the Type III R-M system is responsible for a fewer number of homologous recombination blocks in *Campylobacter jejuni*

**Figure 3:**
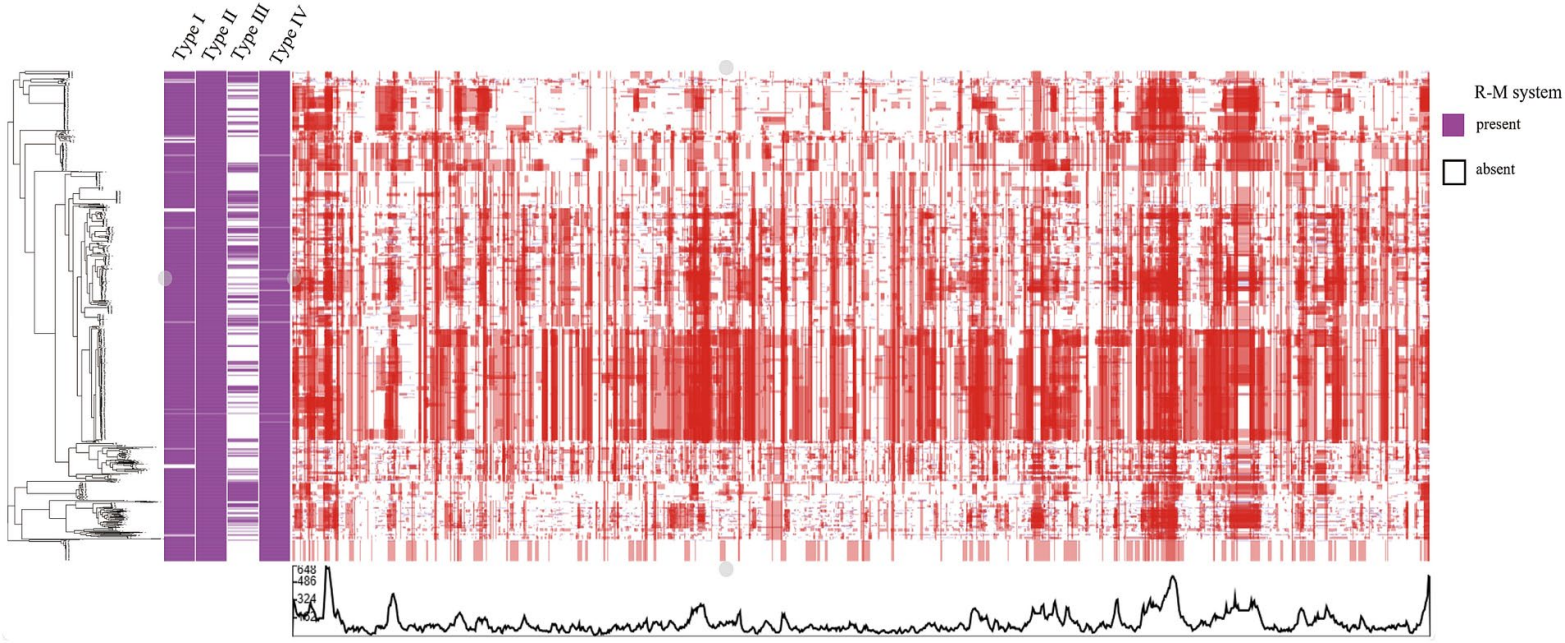
Heatmap of the recombination in *C. jejuni*. The rows in the heatmap represent different *C. jejuni* strain genomes. The blue and red blocks represent recombination events reconstructed on the leaves and internal nodes, respectively.

**Figure 4:**
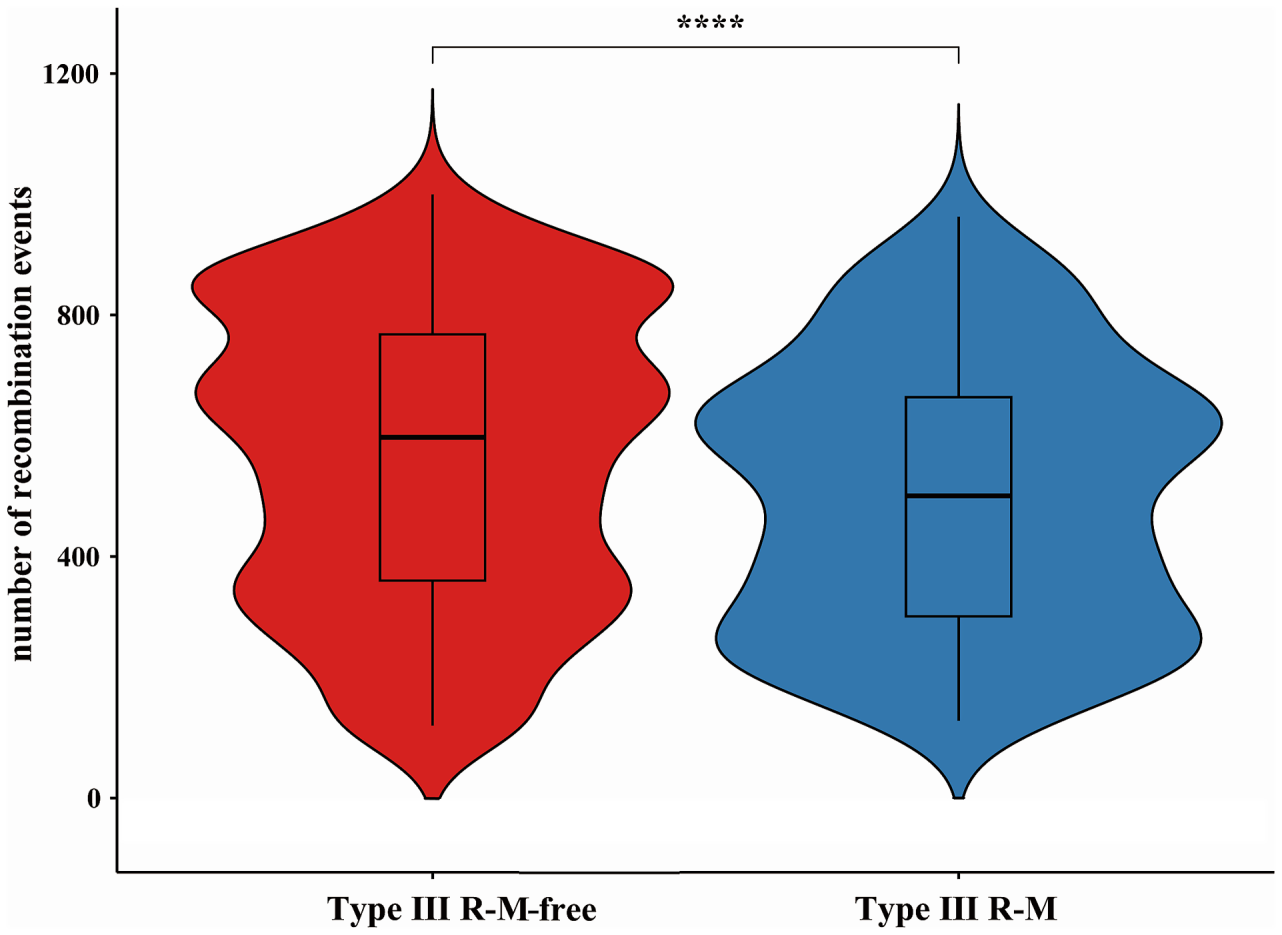
Differences of recombination events in *C. jejuni* with or without the Type III R-M system. The thicker lines in the box plot represent the median. The box plot’s upper and lower boundaries indicate the 75th (Q3) and 25th (Q1) percentiles, respectively. The significance is analyzed using the Wilcoxon-Mann–Whitney test, and the number of asterisks indicates the significant differences: * indicates *p* ≤ 0.05, ** indicates *p* ≤ 0.01, *** indicates *p* ≤ 0.001, **** indicates *p* ≤ 0.0001, and ns indicates no significant difference.

Recombination, as a critical evolutionary force in bacteria, shapes the genomic landscape (Shikov et al., 2023). Using Gubbins, we reconstructed the homologous recombination blocks on the branches of the phylogenetic tree (Figure 3). The total number of the recombination blocks in each *C. jejuni* strain was counted. Through our research, the median number of homologous recombination blocks in the strains possessing the Type III R-M was 500.5, but in the Type III R-M-free strains, the median number increased by 19.38% to 597.5 (Figure 4, Wilcoxon-Mann–Whitney test, *p* < 0.0001), and the covariance value between these two groups was −14.1427, implying a negative correlation between them, which is consistent with the conclusion we have gained in our previous study. This suggests that the presence of the Type III R-M system could reduce the homologous recombination frequency in *C. jejuni*.

### 3.3 The Type III R-M system might have limited effects on the HGT frequency in *Campylobacter jejuni*

**Figure 5:**
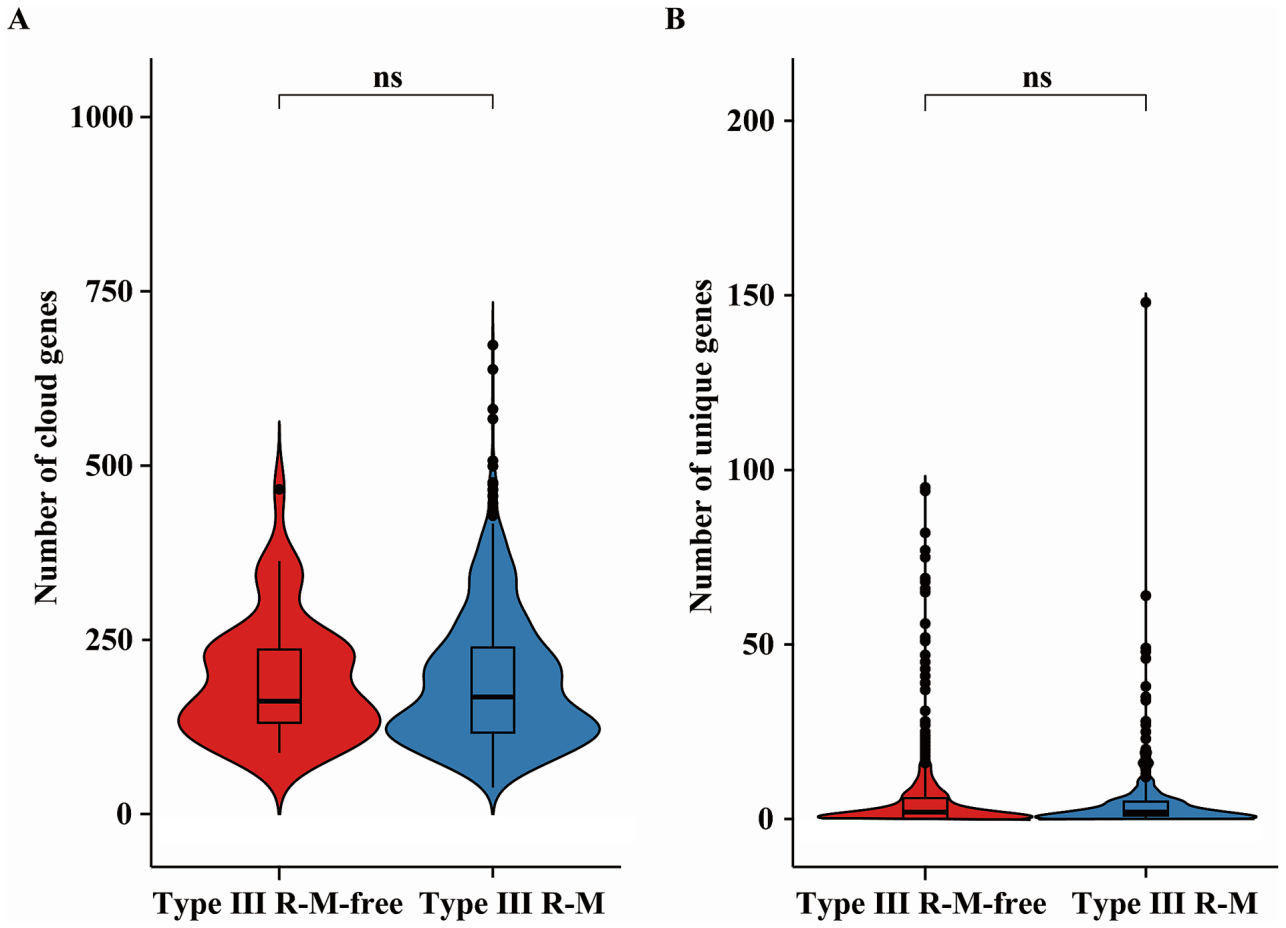
Differences of **(A)** the cloud and **(B)** unique genes in *C. jejuni* with or without the Type III R-M system. The thicker lines in the box plot represent the median. The box plot’s upper and lower boundaries indicate the 75th (Q3) and 25th (Q1) percentiles, respectively. The significance is analyzed using the Wilcoxon-Mann–Whitney test, and the number of asterisks indicates the significant differences: * indicates *p* ≤ 0.05, ** indicates *p* ≤ 0.01, *** indicates *p* ≤ 0.001, **** indicates *p* ≤ 0.0001, and ns indicates no significant difference.

Cloud genes are defined as accessory genes found in 0 ~ 15% ofbacterial species. The HGT frequency of a *C. jejuni* strain can be evaluated by the number of the cloud and unique genes it carries (Xu et al., 2023). We analyzed the distribution of these genes between *C. jejuni* groups with or without the Type III R-M system to explore how the HGT frequency was affected by this system in *C. jejuni* population (Figure 5). Our analysis revealed no significant relationship between the number of cloud and unique genes and the presence of the Type III R-M system (Wilcoxon-Mann–Whitney test, *p* = 0.061 and *p* = 0.41, respectively).

**Figure 6:**
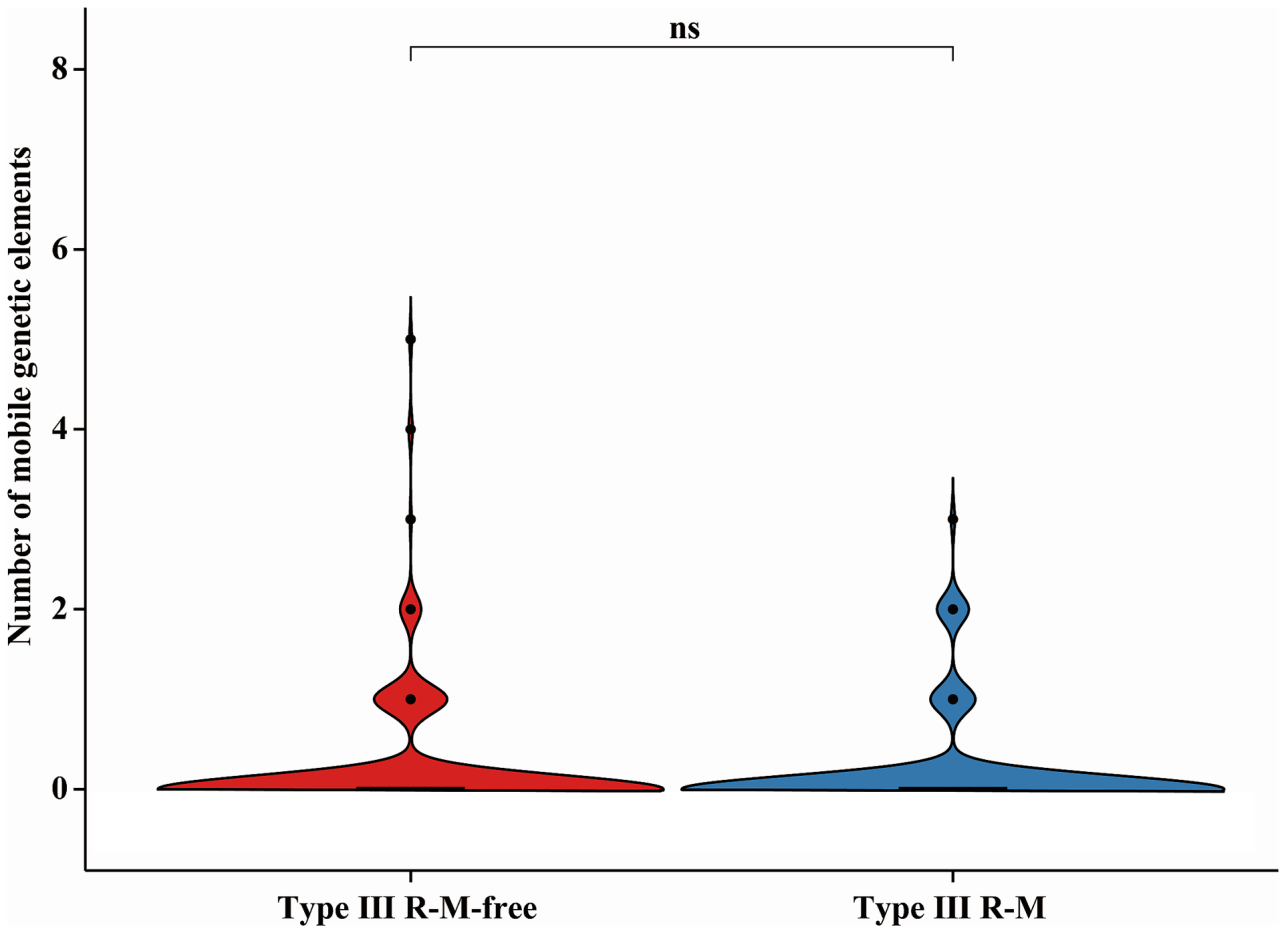
Differences of the MGE in *C. jejuni* with or without the Type III R-M system. The thicker lines in the box plot represent the median. The box plot’s upper and lower boundaries indicate the 75th (Q3) and 25th (Q1) percentiles, respectively. The significance is analyzed using the Wilcoxon-Mann–Whitney test, and the number of asterisks indicates the significant differences: * indicates *p* ≤ 0.05, ** indicates *p* ≤ 0.01, *** indicates *p* ≤ 0.001, **** indicates *p* ≤ 0.0001, and ns indicates no significant difference.

AMR genes carried by MGEs can spread within *C. jejuni* population via HGT (Partridge et al., 2018). We compared the number of the MGEs in *C. jejuni* strains with or without the Type III R-M system to investigate whether the suppression of the acquired AMR gene distribution by Type III R-M system is related to a decline in HGT frequency (Figure 6). The results showed no significant differences in the number of the MGEs between *C. jejuni* groups with or without the Type III R-M system (Wilcoxon-Mann–Whitney test, *p* = 0.13).

In conlusion, our results suggest that the Type III R-M system might have limited effects on HGT frequency in *C. jejuni*.

## 4 Discussion

### 4.1 The possible impact of the R-M system on the transfer of AMR genes in *Campylobacter jejuni*

Recently, *C. jejuni* AMR has become a severe global issue. Although the causes of *C. jejuni* AMR are complex, homologous recombination plays a central in enabling bacteria to acquire AMR elements (Partridge et al., 2018; Shikov et al., 2023). By integrating exogenous genetic materials, bacteria can pre-adapt to changing environment, thereby enhancing their viability. However, since gene acquisition can also lead to gene dysfunction and other side effects, bacteria have evolved ‘defense systems’ to protect their genetic integrity. The restriction-modification (R-M) system is the most prevalent defense mechanism, found in 83% of prokaryotic genomes, which is more than double the incidence of CRISPR-Cas systems (Tesson et al., 2022).

In this study, we first characterized the distribution of R-M systems in *C. jejuni*, revealing an uneven prevalence for both Type I (1,043/1,092, 95.51%) and Type III (334/1,092, 30.59%) systems. Given this disparity, to analyze the impact of R-M systems on AMR gene transmission in *C. jejuni*, we grouped strains based on the presence or absence of Type I and Type III R-M systems and compared the number of AMR genes between these groups. Our analysis revealed no significant differences in AMR gene distribution between strains possessing the Type I R-M system or not (Figure 2A; Wilcoxon-Mann–Whitney test, *p* = 0.59). Conversely, a significant negative correlation was identified between the presence of the Type III R-M system and the number of AMR genes in *C. jejuni* strains (Figure 2B; Wilcoxon-Mann–Whitney test, *p* < 0.0001; covariance = −0.0526). This finding suggests that the Type III R-M system may act as a barrier to AMR gene acquisition. The presence of that system may suppress the transmission of AMR genes by cleaving target exogenous DNA sequences, corresponding to the function of the REases.

### 4.2 The potential underlying mechanisms of the restriction effect on AMR genes transmission by the Type III R-M system in *Campylobacter jejuni*

AMR genes can spread within bacterial populations through homologous recombination, exacerbating the AMR problem (Partridge et al., 2018; Shikov et al., 2023). The macrolide resistance gene *erm (B)* could undergo natural transformation via homologous recombination among *Campylobacter* strains (Wei et al., 2024). Additionally, the horizontally acquired AMR genes could further be transferred via homologous recombination (Gratia, 2017). One study also shows that the horizontal transfer of chromosomal genes via lateral transduction through homologous recombination might provide significant opportunities for the rapid acquisition of virulence factors (Humphrey et al., 2021). To further explore mechanisms underlying the restriction effect on AMR genes transmission by the Type III R-M system, we conducted a series of analyses. Our results show that the presence of the Type III R-M system is associated with a decrease in the number of homologous recombination blocks in *C. jejuni*, strains lacking this system exhibiting a 19.38% higher median number of homologous recombination events (Figure 4; Wilcoxon-Mann–Whitney test, *p* < 0.0001; covariance = −14.1427). Thus, we propose that the Type III R-M system restricts the AMR genes transmission within *C. jejuni* mainly by limiting the frequency of homologous recombination. The homologous recombination frequency of the exogenous AMR genes in *C. jejuni* might be suppressed by the Type III R-M REases, which can cleave DNA sequences and restrict the transmission of AMR genes among *C. jejuni* population, resulting in fewer AMR genes in *C. jejuni* with the Type III R-M system.

Sharing of genes through HGT contributes importantly to the global dissemination of AMR genes, which could be susceptible to bacterial defense systems (Price et al., 2016; Huo et al., 2015). Proposing that the Type III R-M system might influence the acquisition of AMR genes by interfering with the HGT process, we conducted a series of studies. However, results show that there are no significant differences in the number of the cloud or unique genes between *C. jejuni* groups with or without the Type III R-M system (Figure 5; Wilcoxon-Mann–Whitney test, *p* = 0.061 and *p* = 0.41, respectively). MGEs, such as plasmids and the integrative and conjugative elements, disseminating via HGT, have been identified as key vehicles for the dissemination of AMR determinants (Botelho and Schulenburg, 2021). Through the MobileElementFinder software, we found and calculated the number of insertion sequences and unit transposons carried by *C. jejuni* strains with or without the Type III R-M system. Nevertheless, our research shows no significant difference between the number of MGEs in *C. jejuni* with or without that system (Figure 6; Wilcoxon-Mann–Whitney test, *p* = 0.13). Therefore, we suggest that MGEs might have limited correlation with the Type III R-M system in the transfer of AMR genes.

## 5 Conclusion

The analysis of the number distribution of AMR genes between the two groups of *C. jejuni* strains with or without type III R-M system shows that this system might have a restriction effect on AMR gene transfer in *C. jejuni*. Our data indicates that *C. jejuni* without that system seems to appear a higher recombination frequency, but there is no significant difference in the number of unique or cloud genes and MGEs between the two groups. Hence, the type III R-M system may have limited influence on the dissemination of AMR genes between other bacterial species and *C. jejuni*. However, it may restrict the exchange and integration of AMR genes within this species probably by limiting the frequency of homologous recombination to affect the distribution of AMR genes.

The transmission of AMR genes among bacterial populations is a complex process influenced by environmental factors, the genetic characteristics and the bacterial defense systems. This study suggests that the Type III R-M system shows a correlation with the dynamics of the AMR genes transmission between *C. jejuni* strains and explores the possible mechanisms underlying the distribution differences in AMR gene numbers in this species which will be verified through further experiments. Consequently, it provides a theoretical basis for addressing the antibiotic resistance problem in *C. jejuni*.

## Data Availability

The original contributions presented in the study are included in the article/Supplementary material, further inquiries can be directed to the corresponding authors.
